# RNA 5-methylcytosine writer NSUN5 promotes hepatocellular carcinoma cell proliferation *via* a ZBED3-dependent mechanism

**DOI:** 10.1038/s41388-023-02931-z

**Published:** 2024-01-05

**Authors:** Xinyu Gu, Penghui Li, Xiaohui Gao, Yi Ru, Chen Xue, Shujun Zhang, Yafeng Liu, Xinjun Hu

**Affiliations:** 1 Department of Oncology, The First Affiliated Hospital, College of Clinical Medicine, Henan University of Science and Technology, Luoyang, 471000 Henan China; 2 Hepatobiliary Pancreatic Surgery, The First Affiliated Hospital, College of Clinical Medicine, Henan University of Science and Technology, Luoyang, 471000 Henan China; 3 Department of Infectious Diseases, The First Affiliated Hospital of Zhengzhou University, Zhengzhou, 450052 Henan China; 4 Department of Infectious Diseases, The First Affiliated Hospital, College of Clinical Medicine, Henan University of Science and Technology, Luoyang, 471000 Henan China

**Keywords:** Cancer, Diagnostic markers

## Abstract

Hepatocellular carcinoma (HCC) is one of the leading contributors to cancer-related mortality worldwide. Nop2/Sun domain family member 5 (NSUN5), a conserved RNA 5-methylcytosine methyltransferase, is conventionally recognized as oncogenic. However, its role in HCC development remains unknown. In this study, we observed a remarkable upregulation of NSUN5 expression in both tumor tissues from patients with HCC, establishing a correlation with unfavorable clinical outcomes. NSUN5 knockdown and overexpression significantly inhibited and promoted HCC cell proliferation, respectively. Additionally, employing a combination of methylated RNA immunoprecipitation sequencing (MeRIP-seq) and RIP-seq techniques, we identified zinc finger BED domain-containing protein 3 (ZBED3) as a novel downstream target of NSUN5. Additionally, we found that the overexpression of ZBED3 counteracted the tumor-suppressing effect of NSUN5 knockdown and simultaneously reversed the inhibition of the Wnt/β-catenin signaling pathway. In summary, we elucidated the oncogenic role of NSUN5 in HCC development and identified the ZBED3/Wnt/β-catenin signaling pathway as its downstream target. This study provides a novel therapeutic target for further development in HCC treatment.

## Introduction

Liver malignancies are among the most prevalent types of cancer worldwide. Hepatocellular carcinoma (HCC), the predominant pathological subtype, accounts for over 80% of all liver cancer cases [1, 2] and ranks as the third leading cause of cancer-related fatalities [3, 4]. Established risk factors for HCC include chronic viral hepatitis caused by hepatitis B or hepatitis C viruses, as well as alcohol-related liver diseases. Recently, nonalcoholic fatty liver disease and obesity have also emerged as significant contributors to HCC development [5, 6]. Given its high incidence and mortality rates, understanding the molecular mechanisms governing HCC cell proliferation is imperative for informing the development of novel antitumor therapies.

Post-transcriptional RNA modifications, including methylation, acetylation, uridylation, and pseudouridine (Ψ) formation, have been extensively studied since their initial discovery in the 1950s [7, 8]. Proteins involved in this biological process can be categorized into three distinct categories: “writers” and “erasers,” which add and remove moieties at RNA modification sites, respectively, and “readers,” which specifically recognize and bind to the modified RNA sites. The interplay among these writers, erasers, and readers modulates the proper functioning of RNA [9].

Among RNA methylation modifications, the N^6^-methyladenosine modification stands out as the most extensively investigated, while the 5-methylcytosine (m^5^C) modification is gaining increasing attention due to its emerging roles in various biological processes, including tumorigenesis [10, 11]. Nop2/Sun domain family member 5 (NSUN5) primarily functions as a conserved RNA m^5^C methyltransferase, or m^5^C writer [12]. Over the past decade, the oncogenic role of NSUN5 has been identified in several cancer types. In gastric cancer, NSUN5 modulates ferritin heavy chain 1 and inhibits ferroptosis, ultimately enhancing tumor cell proliferation [13]. In clear-cell renal cell carcinoma, NSUN5 overexpression facilitates cancer cell invasion, proliferation, and migration while also inhibiting apoptosis by suppressing the p53 signaling pathway [14]. In colorectal cancer, NSUN5 acts as a tumor promoter by regulating the cell cycle [15]. Contrary to these findings, Maxime et al. reported tumor-suppressing effects of NSUN5 in in vivo glioma models. The knockdown of NSUN5 results in the loss of methylation at the C3782 position of 28S rRNA, leading to an overall decline in protein synthesis and tumor cell proliferation [16]. Nevertheless, the precise role of NSUN5 in HCC development remains incompletely understood. Recent findings demonstrate that NSUN5 is upregulated in HCC tissues [17] and may promote HCC progression by enhancing gene translation [18]. However, the underlying mechanisms require further investigation.

In this study, we found that NSUN5 expression is elevated in tumor tissues compared with normal liver tissues of patients with HCC. Moreover, we demonstrated that its knockdown substantially inhibits HCC cell proliferation. We integrated methylated RNA immunoprecipitation sequencing (MeRIP-seq) data with transcriptomics data to unveil the m^5^C modification profiles of NSUN5-knockdown cells. Subsequently, we identified zinc finger BED domain-containing protein 3 (ZBED3) as a novel target of NSUN5. In summary, we elucidated a novel NSUN5/m^5^C/ZBED3 axis through which NSUN5 promotes HCC development. Our study elucidates a promising therapeutic target for HCC treatment.

## Results

### High NSUN5 expression was associated with tumor progression in patients with HCC

**Fig. 1:**
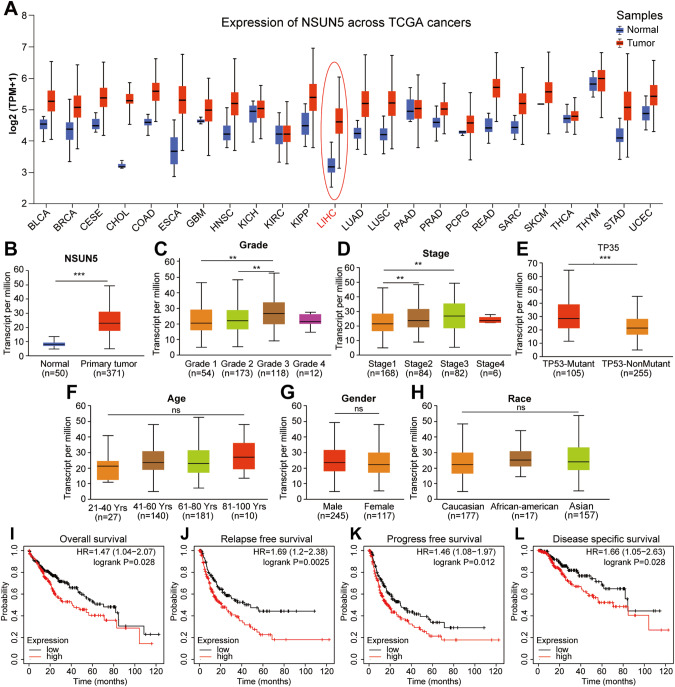
High NSUN5 is associated with tumor progression in patients with HCC. **A** Pan-cancer analysis utilizing patient samples from TCGA database showed dysregulated NSUN5 expression in various cancer types, including HCC. **B** In patients with HCC, NSUN5 demonstrated significant upregulation in primary tumor tissues (*n* = 371) compared with normal tissues (*n* = 50). **C** NSUN5 exhibited significant upregulation in tumors with higher grades, with Grade 4 tumors showing a slight decrease in NSUN5 transcription (Grade 3 vs. Grade 1; Grade 3 vs. Grade 2). **D** NSUN5 expression significantly increased with the stage of HCC, except in Stage 4 HCC (Stage 2 vs. Stage 1; Stage 3 vs. Stage 1). **E** NSUN5 expression was higher in patients with TP53 gene mutations. **F**–**H** No significant correlations were observed between NSUN5 expression and patient age, gender, or race. **I**–**L** Patients with HCC exhibiting high NSUN5 expression experienced relatively poorer survival outcomes than those exhibiting low NSUN5 expression, as indicated by OS (HR = 1.47 [1.04–2.07], log-rank *p* = 0.028), RFS (HR = 1.69 [1.2–2.38], log-rank *p* = 0.0025), PFS (HR = 1.46 [1.08–1.97], log-rank *p* = 0.012), and DSS (HR = 2.13 [1.05–2.63], log-rank *p* = 0.028).

A pan-cancer analysis of NSUN5 transcription, utilizing patient samples sourced from the Cancer Genome Atlas (TCGA) database, revealed dysregulation of its expression across various cancer types, including HCC (Fig. 1A). In patients with HCC, NSUN5 exhibited significant upregulation in primary tumor tissues (*n* = 371) compared with normal liver tissues (*n* = 50, Fig. 1B).

The prognostic significance of NSUN5 expression in patients with HCC was explored by examining its association with clinical parameters, including tumor grade, stage, TP53 mutation status, age, gender, and race. NSUN5 transcription exhibited a significant upregulation in tumors of higher grades, except for Grade 4 tumors, which showed a slight decrease in NSUN5 transcription (Grade 3 vs. Grade 1; Grade 3 vs. Grade 2; Fig. 1C). Similarly, NSUN5 expression increased markedly with HCC stage, except for Stage 4 (Stage 2 vs. Stage 1; Stage 3 vs. Stage 1; Fig. 1D). The inconsistent results observed for Grade 4 and Stage 4 HCC may be attributed to limited clinical sample availability. Moreover, NSUN5 transcription was more likely to be elevated in patients with TP53 mutations (Fig. 1E). No significant correlations were found between NSUN5 expression and patient age, gender, or race (Fig. 1F–H).

Kaplan–Meier survival analysis was performed to evaluate the prognostic significance of NSUN5 expression in HCC. Patients were stratified into high- and low-expression groups based on the median NSUN5 expression value. Patients in the high-expression group exhibited relatively poorer survival outcomes compared to those in the low-expression group across various survival metrics, including overall survival (OS; hazard ratio [HR] = 1.47 [1.04–2.07], log-rank *p* = 0.028), recurrence-free survival (RFS; HR = 1.69 [1.2–2.38], log-rank *p* = 0.0025), progression-free survival (PFS; HR = 1.46 [1.08–1.97], log-rank *p* = 0.012), and disease-specific survival (DSS; HR = 2.13 [1.05–2.63], log-rank p = 0.028) (Fig. 1I–L). Taken together, these results indicate that elevated NSUN5 expression in patients with HCC plays a crucial role in tumor progression and holds promise as a prognostic marker.

### NSUN5 expression levels regulated HCC cell proliferation and viability in vitro

**Fig. 2:**
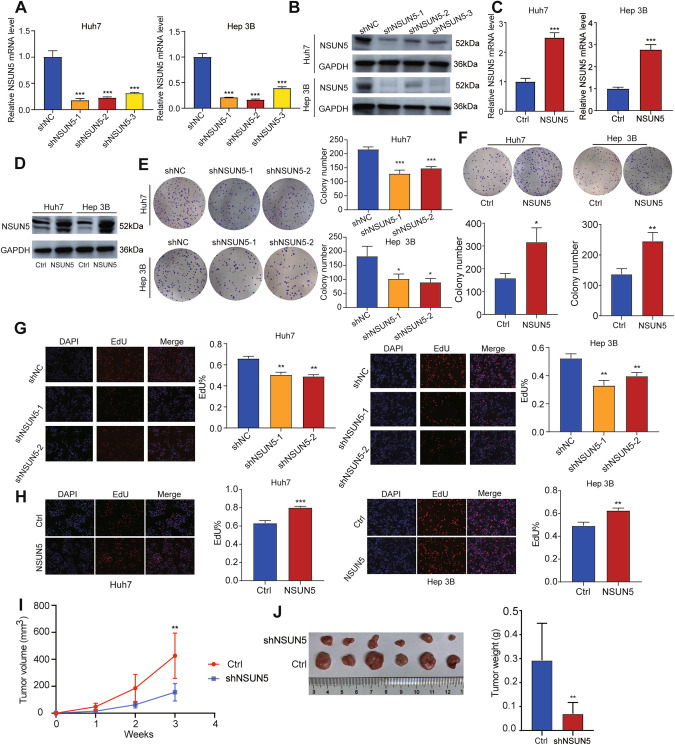
NSUN5 promotes the proliferation of HCC cells in vitro. **A**, **B** Significant downregulation of NSUN5 was confirmed through qRT–PCR and western blot analysis following shRNA treatment. **C**, **D** Effective overexpression of NSUN5 was validated through qRT–PCR and western blot analysis. **E**, **G** Knockdown of NSUN5 using shNSUN5-1 and shNSUN5-2 critically impaired the proliferative viability of both Huh7 and Hep 3B cells. **F**, **H** NSUN5 overexpression significantly promoted the proliferation of both Huh7 and Hep 3B cells. **I**, **J** Tumor size and weight in tumor-bearing mice are presented.

To investigate the impact of NSUN5 expression at the cellular level, we performed loss-of-function and gain-of-function experiments. We employed three short hairpin RNAs (shRNAs; shNSUN5-1, shNSUN5-2, and shNSUN5-3) to knockdown endogenous NSUN5 expression in Huh7 and Hep 3B cells. Subsequently, shNSUN5-1 and shNSUN5-2 were selected for subsequent experiments owing to their superior knockdown efficacy in both cell lines, as confirmed by Quantitative real time polymerase chain reaction(qRT–PCR) and western blot analysis (Fig. 2A, B). Similarly, NSUN5 overexpression in these cell lines *via* lentiviral transduction was assessed through qRT–PCR and western blot analysis (Fig. 2C, D). Suppression of NSUN5 critically compromised the proliferative capacity of both cell lines, as determined by colony formation (Fig. 2E). Conversely, NSUN5 overexpression significantly enhanced tumor cell proliferation (Fig. 2F). The results of 5-ethynyl-2′-deoxyuridine (EdU) assays were consistent with those of colony formation assays (Fig. 2G, H). Subsequently, stable shNegativeControl (shNC) cells and stable shNSUN5 cells were injected into nude mice. After three weeks post-injection, we found that the mice receiving stable shNSUN5 cells developed tumors of smaller size and lighter weight compared to those receiving shNC cells (Fig. 2I, J). These results indicate that NSUN5 promotes the growth of HCC cells in vivo.

### Potential targets of NSUN5 were identified through m^5^C-MeRIP-seq

**Fig. 3:**
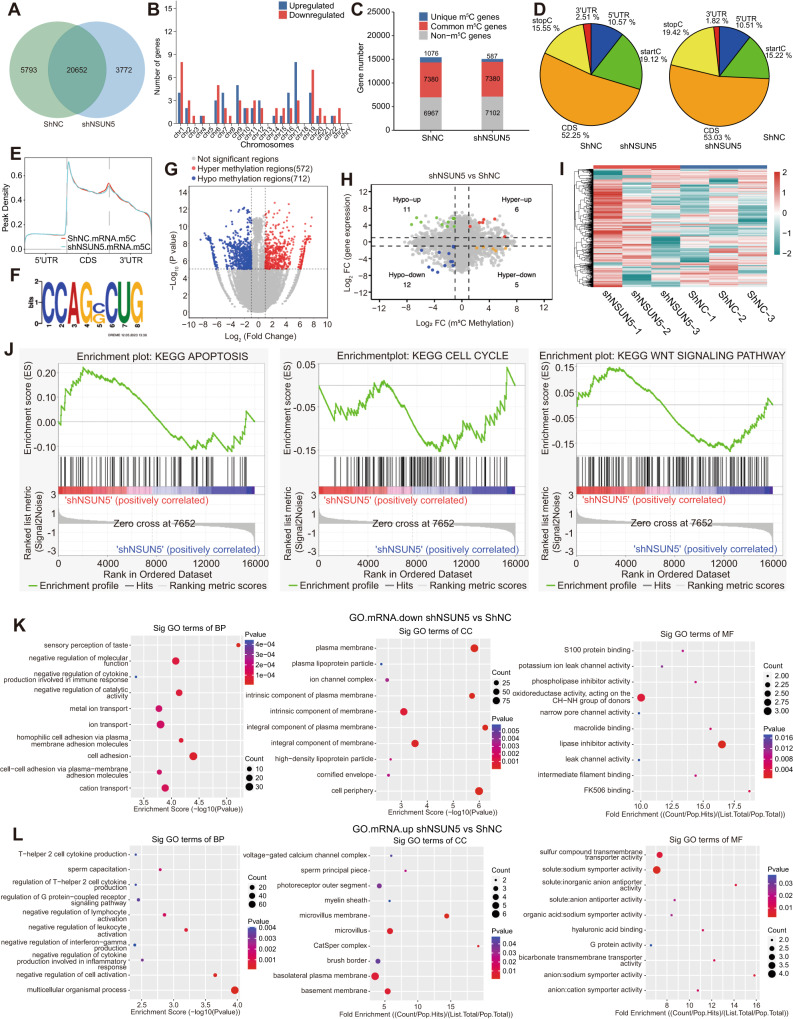
Results of m^5^C-MeRIP-seq of mRNAs in Huh7 cells with and without NSUN5 knockdown. **A** NSUN5 knockdown led to 3772 hypermethylated and 5793 hypomethylated sits. **B** Chr17 exhibited the highest number of upregulated genes, while Chr1 exhibited the highest number of downregulated genes. No significant alterations in gene expression were observed on Chr5, Chr13, Chr18, and ChrY after NSUN5 knockdown. **C** Transcriptome-wide methylation profiling of expressed RNAs revealed 1076 unique m^5^C modified genes in shNC-treated Huh7 cells and 587 m^5^C modified genes in shNSUN5-treated Huh7 cells, with 7380 genes displaying common m^5^C modified genes. **D**–**F** In both shNC- and shNSUN5-treated Huh7 cells, m^5^C modifications were predominantly located within the CDS and enriched near the start and stop codons. The most conserved motif was CCAGRCUG (R = C/G). **G** A volcano plot depicts 572 hypermethylated and 712 hypomethylated genes in shNSUN5-treated Huh7 cells compared to shNC-treated cells. **H** A four-quadrant diagram illustrates six genes that were hypermethylated and upregulated (hyper-up), five that were hyper-down, eleven that were hypo-up, and twelve that were hypo-down following NSUN5 knockdown. **I** A heatmap displays the differential methylation patterns of mRNAs following NSUN5 knockdown. **J** GSEA of NSUN5-associated DEGs indicated that NSUN5 plays a role in cell cycle regulation, apoptosis, and the Wnt signaling pathway. **K**, **L** GO enrichment analyses of cells with NSUN5 knockdown and shNC showed that NSUN5-associated DEGs were associated with cell adhesion, taste perception, negative regulation of cell activation, multicellular organismal processes, etc.

We performed m^5^C-MeRIP-seq analysis at the transcriptional level using Huh7 cells. NSUN5 knockdown drastically altered the mRNA methylation profile of Huh7 cells. A total of 26,445 and 24,424 m^5^C sites of mRNA were found in sh-NC and sh-NSUN5 Huh7 cells, respectively, and the two groups shared 20,652 m^5^C sites (Fig. 3A). Moreover, Chromosome 17 exhibited the highest number of upregulated genes, while Chr1 displayed the most downregulated genes. No significant changes in gene expression were observed on Chr5, Chr13, Chr18, and ChrY following NSUN5 knockdown (Fig. 3B).

Subsequently, we examined transcriptome-wide methylation patterns in Huh7 cells treated with shNC or shNSUN5. We identified a total of 1,076 unique m^5^C modified genes in the shNC group and 587 in the shNSUN5 group, with 7380 m^5^C modified genes being common to both (Fig. 3C). In both cell groups, these m^5^C modifications were predominantly situated within the coding sequences (CDS; Fig. 3D) and exhibited enrichment near the start and stop codons (Fig. 3E). The most highly conserved motif identified was CCAGRCUG (R = C/G; Fig. 3F). To visually represent the changes in methylation in shNSUN5-treated Huh7 cells in comparison to shNC-treated cells, we employed a volcano plot, revealing 572 hypermethylated and 712 hypomethylated genes (Fig. 3G). A four-diagram chart was prepared by combining the alterations in both methylation and expression profiles within the two cell groups. In total, six hypermethylated and upregulated (hyper-up), five hyper-down, eleven hypo-up, and twelve hypo-down genes were identified and subjected to further analysis (Fig. 3H). To illustrate the differential methylation of mRNAs in shNSUN5- and shNC-treated Huh7 cells, we generated a heatmap (Fig. 3I). Gene set enrichment analysis (GSEA) of differentially expressed genes (DEGs) associated with NSUN5 suggested its involvement in cell cycle regulation, apoptosis, and Wnt signaling (Fig. 3J). Furthermore, gene ontology (GO) enrichment analysis of cells with NSUN5 knockdown and control revealed that the DEGs associated with NSUN5 were linked to cell adhesion, taste perception, negative regulation of cell activation, multicellular organismal processes, etc. (Fig. 3K, L).

**Fig. 4:**
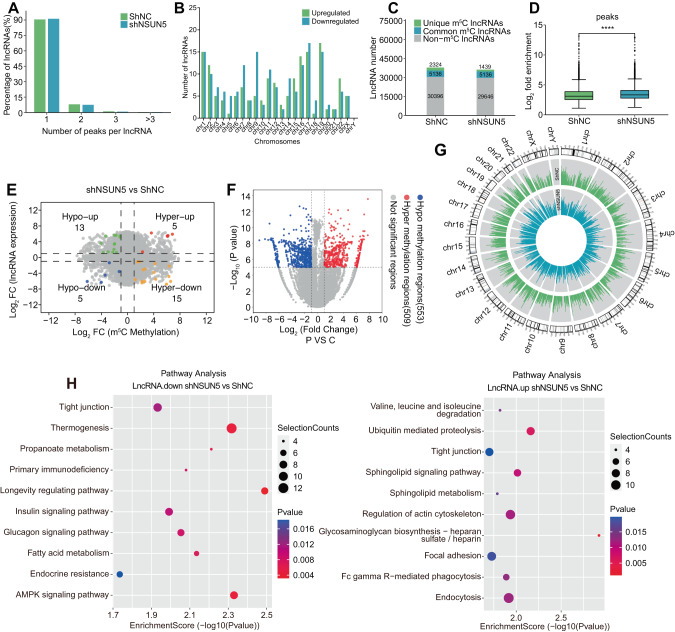
Results of m^5^C-MeRIP-seq of lncRNAs in Huh7 cells with and without NSUN5 knockdown. **A** Most lncRNAs in both shNC- and shNSUN5-treated Huh7 cells exhibited a single m^5^C methylation peak. **B** Chromosomal distribution of upregulated and downregulated lncRNAs. Chr19 displayed the highest number of upregulated lncRNAs, while Chr17 exhibited the highest number of downregulated lncRNAs. **C** In shNC- and shNSUN5-treated Huh7 cells, 2324 and 1439 unique m^5^C-methylated lncRNAs were identified, along with 30,396 and 29,646 non-methylated lncRNAs, respectively. Additionally, 5136 m^5^C-methylated lncRNAs were common to both groups. **D** The lncRNA methylation peaks in the two cell groups exhibited a significant difference in fold enrichment. **E** A four-quadrant diagram illustrates five hyper-up, fifteen hyper-down, thirteen hypo-up, and five hypo-down lncRNAs following NSUN5 knockdown. **F** A volcano plot displays 509 hypermethylated and 553 hypomethylated lncRNA regions following NSUN5 knockdown. **G** A Circos map visually presents the chromosome locations and fold enrichment of m^5^C methylation peaks in both shNC- and shNSUN5-treated Huh7 cells. **H** Pathway analysis indicated that lncRNAs associated with NSUN5 knockdown were preferentially enriched in pathways associated with longevity regulation, AMPK signaling, glycosaminoglycan biosynthesis (heparan sulfate/heparin), and ubiquitin-mediated proteolysis.

Moreover, we performed m^5^C-MeRIP-seq of long non-coding RNAs (lncRNAs) and circular RNAs (circRNAs). Most lncRNAs exhibited a single m^5^C methylation peak in both shNC- and shNSUN5-treated Huh7 cells (Fig. 4A). Chr19 exhibited the highest number of upregulated lncRNAs, while Chr17 exhibited the greatest number of downregulated genes (Fig. 4B). A total of 2324 and 1439 unique m^5^C-methylated lncRNAs were identified in shNC- and shNSUN5-treated Huh7 cells, respectively, with 5136 lncRNAs being common to both conditions (Fig. 4C). The fold enrichment of lncRNA methylation peaks significantly differed between these two groups of cells (Fig. 4D). Subsequently, we created a four-quadrant diagram to illustrate five hyper-up, fifteen hyper-down, thirteen hypo-up, and five hypo-down lncRNAs (Fig. 4E). A volcano plot was employed to visualize 509 hypermethylated and 553 hypomethylated lncRNA regions following NSUN5 knockdown (Fig. 4F). Employing a previously published protocol [19], we generated a Circos map to visualize the chromosome locations and fold enrichment of m^5^C methylation peaks in both cell groups (Fig. 4G). Finally, pathway analysis indicated that differently expressed lncRNAs associated with NSUN5 were preferentially enriched in longevity regulation, AMPK-activated protein kinase signaling, glycosaminoglycan biosynthesis (heparan sulfate/heparin), and ubiquitin-mediated proteolysis (Fig. 4H). The results of the m^5^C-MeRIP-seq analysis of circRNAs are illustrated in Supplementary Fig. 1.

**Fig. 5:**
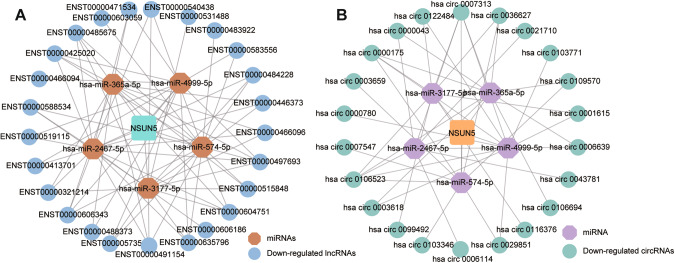
CeRNA networks for NSUN5 based on transcription profiles of shNC- and shNSUN5-treated Huh7 cells. **A** A ceRNA network integrating NSUN5, miRNAs, and lncRNAs. **B** ceRNA network integrating NSUN5, miRNAs, and circRNAs.

Moreover, we constructed a competing endogenous RNA (ceRNA) network incorporating NSUN5, microRNAs (miRNAs), and lncRNAs based on the transcription profiles of shNC- and shNSUN5-treated Huh7 cells to consolidate potential regulatory interactions among these molecules. Validated miRNAs, such as hsa-miR-574-5p and hsa-miR-365a-5p, were integrated into the network. The establishment of the network offers potential targets for exploring the mechanisms underlying NSUN5-mediated tumorigenesis (Fig. 5A). Similarly, we employed analogous methods to predict a ceRNA network involving NSUN5, miRNAs, and circRNAs (Fig. 5B).

### Analysis of genes binding to the NSUN5 protein through RIP-seq

**Fig. 6:**
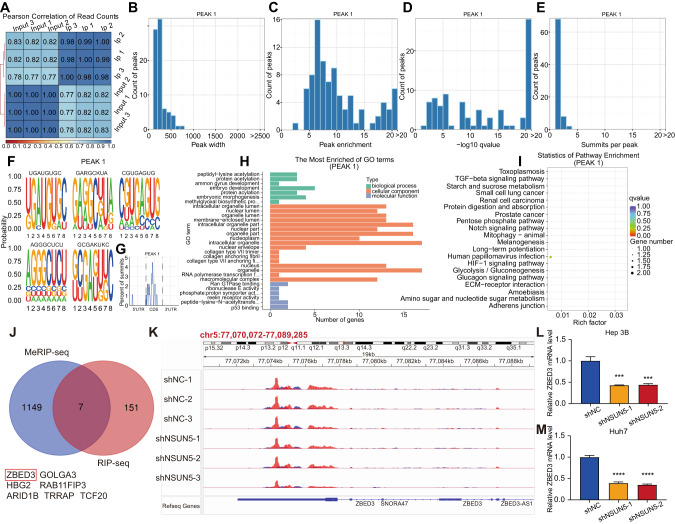
Results of RIP-seq of the Peak-1 group in Huh7 cells and identification of *ZBED3* as the target gene of NSUN5. **A** Satisfactory correlation coefficients were observed among different sample groups. **B** The Peak 1 group primarily consisted of RNAs with widths ranging from 100 to 300 nt, with no RNAs exceeding 800 nt in width. **C** Peak enrichment analysis showed that most of the RNAs in the Peak-1 group exhibited total read counts between 5 and 10. **D** Significance levels among different RNA peaks displayed a scattered distribution in general. **E** Nearly all RNA peaks exhibited a single summit, with only a few displaying two to three summits. **F** The top five most significant mRNA motifs are illustrated. **G** The peaks were enriched in the CDS of mRNAs. A relatively lower number of gene peaks were identified in the 5′-UTR of mRNAs, while no gene peaks were found in the 3′-UTR. **H** GO enrichment analysis showed that peak-overlapping genes in the Peak-1 group were enriched in biological processes such as embryonic development, cellular components, including intracellular organelles, and molecular functions associated with binding to Ran GTPase. **I** KEGG enrichment analysis indicated significant enrichment of peak-overlapping genes in the Peak-1 group in pathways such as the pentose phosphate pathway, starch and sucrose metabolism, amino sugar and nucleotide sugar metabolism, and the Notch signaling pathway. **J** Overlapping the 1158 DEGs identified by MeRIP-seq and the 158 genes exhibiting high binding affinities for NSUN5 identified by RIP-seq revealed seven potential target genes: *ZBED*3, *GOLGA3*, *HBG2*, *RAB11FIP3*, *ARID1B*, *TRRAP*, and *TCF20*. **K** Knockdown of NSUN5 significantly reduced m^5^C modification levels in *ZBED3* in Huh7 cells. **L** NSUN5 deficiency markedly suppressed ZBED3 expression in Hep 3B cells. **M** NSUN5 deficiency significantly suppressed ZBED3 expression in Huh7 cells.

We aimed to identify RNAs exhibiting a high affinity for the NSUN5 protein through RIP-seq analysis of Huh7 cells. The cellular lysate, denoted as “Input,” was immunoprecipitated using the NSUN5 protein to isolate NSUN5-bound RNAs designated as “IP.” Huh7 cells were divided into three identical groups, and these groups were analyzed concurrently to guarantee the stability and reproducibility of the results. A high correlation was observed among the Input and IP groups, with a coefficient approaching 1.00 (Fig. 6A).

Subsequently, we compared the gene expression level between the Input and IP groups using MACS2 for peak calling (threshold *q* value = 0.05) [20]. In each paired Input and IP group, we identified a set of genes referred to as “Peak” by assessing differences in gene expression. We identified 78, 67, and 98 gene peaks in the “Peak-1,” “Peak-2,” and “Peak-3” groups, respectively. The number of gene summits slightly exceeded that of gene peaks, owing to the merging of a few summits into a common peak. Consequently, a total of 90, 74, and 109 gene summits were identified in the respective three groups.

We analyzed the gene peaks, considering parameters such as peak width (the length of the protein-binding sequence), enrichment level, statistical significance, number of summits, and motif characteristics. In the Peak-1 group, the majority of RNAs exhibited widths ranging from 100 to 300 nt, with a few instances of wider RNA peaks, none of which exceeded 800 nt (Fig. 6B). Peak enrichment analysis showed that most RNAs in the Peak-1 group exhibited between 5 and 10 total reads (Fig. 6C). The significance levels across different RNA peaks displayed a dispersed distribution without clear clustering patterns (Fig. 6D). Moreover, almost all RNA peaks exhibited a single summit, with only a limited number displaying two to three summits (Fig. 6E). To identify the top five most significant mRNA motifs, we employed HOMER (Fig. 6F). Similar patterns were observed in the Peak-2 and Peak-3 groups (Supplementary Figs. 2A–E and 3A–E), reinforcing the robustness and consistency of our RIP-seq analysis.

The gene peaks were enriched in the CDS of mRNAs in all three groups. A comparatively lower number of peaks were detected in the 5′-untranslated region (UTR), with no peaks observed in the 3′-UTR (Fig. 6G; Supplementary Fig. 2F and 3F).

Subsequently, we performed GO and Kyoto Encyclopedia of Genes and Genomes (KEGG) enrichment analyses of genes that overlapped with at least one peak at any position, referred to as peak-overlapping genes. In the Peak-1 group, these peak-overlapping genes were enriched in biological processes such as embryonic development, cellular components, including intracellular organelles, and molecular functions involving the binding of Ran GTPase (Fig. 6H). Furthermore, KEGG enrichment analysis indicated a significant enrichment of peak-overlapping genes in the Peak-1 group in pathways such as the pentose phosphate pathway, starch and sucrose metabolism, amino sugar and nucleotide sugar metabolism, as well as the Notch signaling pathway (Fig. 6I). Similar results from GO and KEGG enrichment analyses for the Peak-2 and Peak-3 groups can be found in Supplementary Fig. 2G and 2H and Supplementary Fig. 3G and 3H, respectively.

### NSUN5 targeted the *ZBED3* gene

As previously mentioned, we conducted m^5^C-MeRIP-seq of NSUN5-associated DEGs, enabling precise identification of key target genes influencing this process. We compared 1,158 DEGs identified *via* MeRIP-seq with 158 genes exhibiting high binding affinity for NSUN5, as revealed by RIP-seq. This analysis yielded seven overlapping target genes: *ZBED3*, golgin A3 (*GOLGA3)*, hemoglobin subunit gamma 2 (*HBG2)*, RAB11 family interacting protein 3 (*RAB11FIP3)*, AT-rich interaction domain 1B(*ARID1B)*, transformation/transcription domain associated protein (*TRRAP)*, and transcription factor 20 (*TCF20*) (Fig. 6J). Among these candidates, *ZBED3* has been previously associated with tumorigenesis. ZBED3, which positively regulates Wnt/β-catenin signaling by enhancing β-catenin expression, thereby promoting the proliferation and invasiveness of lung cancer, was selected for subsequent experimentation.

NSUN5 knockdown significantly reduced the m^5^C modification of *ZBED3* in Huh7 cells (Fig. 6K). Moreover, NSUN5 deficiency significantly suppressed ZBED3 expression in both Hep 3B (Fig. 6L) and Huh7 cells (Fig. 6M). Taken together, these findings suggest that ZBED3 operates downstream of NSUN5 and plays a pivotal role in HCC development.

Considering all the results, we propose that NSUN5 contributes to HCC development by modulating its target gene, *ZBED3*, *via* m^5^C modification.

### ZBED3 overexpression rescued the tumor-suppressive effect induced by NSUN5 degradation in HCC cells

**Fig. 7:**
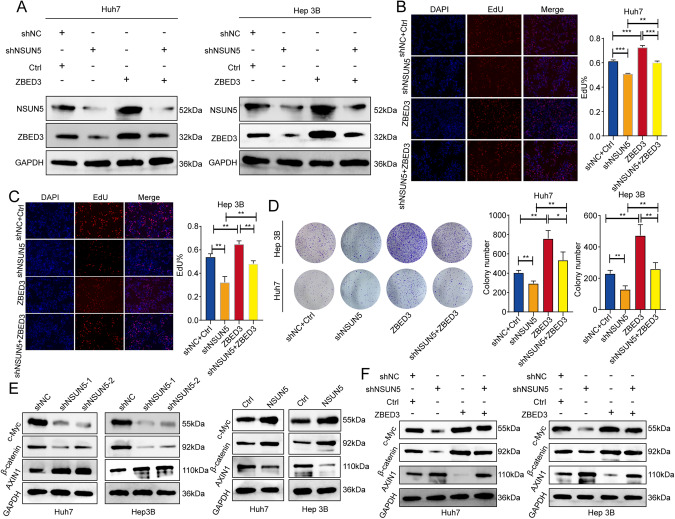
ZBED3 overexpression counteracted the tumor-suppressive effect induced by NSUN5 silencing in HCC cells. **A** Western blotting results for lysates of Huh7 and Hep 3B cells subjected to shNC or shNSUN5 transfection, ZBED3 overexpression, and/or ZBED3 overexpression control group. **B**, **C** EdU assays of the aforementioned four groups of Huh7 and Hep 3B cells. **D** Colony formation assays of the aforementioned four groups of Huh7 and Hep 3B cells. **E** Western blotting results of c-Myc, β-catenin, and AXIN1 in lysates of Huh7 and Hep 3B cells subjected to NSUN5 knockdown and overexpression. **F** Western blotting results of c-Myc, β-catenin, and AXIN1 in lysates of the aforementioned four groups of Huh7 and Hep 3B cells.

To further investigate the role of ZBED3 in NSUN5-related tumorigenesis, we determined whether ZBED3 overexpression affected HCC cell proliferation under NSUN5 deficiency. We established stable NSUN5 knockdown in both Huh7 and Hep 3B cells using shRNAs (Fig. 7A). As expected, NSUN5 deficiency significantly reduced ZBED3 expression levels and substantially inhibited the proliferation of both Huh7 (Fig. 7B) and Hep 3B cells (Fig. 7C). In addition, ZBED3 overexpression almost completely reversed the tumor-suppressive effect induced by NSUN5 knockdown. Moreover, colony formation assays showed similar results (Fig. 7D), collectively indicating that ZBED3 plays an indispensable and positive role in NSUN5-associated tumorigenesis.

Considering NSUN5-related DEGs are enriched in the Wnt pathway and the active involvement of ZBED3 in the Wnt/β-catenin signaling pathway, we subsequently investigated whether NSUN5 knockdown and overexpression affected the expression levels of c-Myc, β-catenin, and AXIN1. Previous studies have shown that AXIN1 participates in the phosphorylation and degeneration of β-catenin, which suppresses c-Myc expression and activation of downstream signaling molecules [21]. As expected, NSUN5 deficiency significantly decreased the concentration of c-Myc and β-catenin while simultaneously promoting AXIN1 expression (Fig. 7E). In contrast, NSUN5 overexpression in HCC cells yielded contrasting results (Fig. 7E).

Furthermore, we investigated the impact of ZBED3 overexpression on the Wnt/β-catenin signaling pathway. ZBED3 overexpression effectively counteracted the alterations observed in the expression of c-Myc, β-catenin, and AXIN1 resulting from NSUN5 knockdown in both Huh7 and Hep 3B cells (Fig. 7F). Collectively, these results provide additional confirmation for our hypothesis that NSUN5 promotes HCC proliferation through its positive modulation of the ZBED3 and β-catenin signaling pathways.

## Discussion

Aberrant m^5^C modifications of RNA have been reported to be closely associated with tumorigenesis and immune infiltration across various cancer types, rendering them promising targets for anticancer therapy. Recently, prognostic models based on m^5^C-associated genes have emerged for HCC, offering novel insights into the role of this RNA modification in cancer development [22–26]. A previous study revealed that elevated NSUN5 expression promotes both in vitro HCC cell proliferation and migration as well as in vivo HCC tumor growth. The researchers hypothesized that NSUN5 facilitates tumor growth by enhancing protein translation [18], although the precise molecular mechanisms remained elusive. Hence, we conducted this study to elucidate potential downstream molecules and pathways regulated by NSUN5.

In this study, we initially observed elevated NSUN5 expression in both TCGA-LIHC (liver hepatocellular carcinoma) cohort. NSUN5 exhibited upregulation in many cancer types, indicating its potential role as a significantly oncogenic factor. Furthermore, we observed associations between NSUN5 expression and clinical parameters, such as tumor grade, stage, and TP53 mutation status. Kaplan–Meier analysis indicated promising prognostic implications. However, further evidence is needed to corroborate these findings, given that the data used in this study were limited. Moreover, we demonstrated that NSUN5 knockdown critically impairs the proliferative viability of HCC cells and vice versa. Overall, our findings prove that NSUN5 serves as a tumor-promoting gene in HCC.

To elucidate the molecular mechanisms underlying NSUN5-mediated HCC proliferation, we performed m^5^C-MeRIP-seq and RIP-seq analyses to identify potential downstream molecules regulated by NSUN5. m^5^C modification sites were predominantly situated within the CDS and exhibited proximity to both the start and stop codons. Following NSUN5 knockdown, we identified genes exhibiting altered expression and/or methylation levels. Through the integration of m^5^C-MeRIP-seq and RIP-seq results, we identified seven target genes displaying altered expression levels and exhibiting high binding affinities for NSUN5. Among these candidates, *ZBED3* was subjected to subsequent analysis. NSUN5 knockdown significantly diminished the expression levels and m^5^C modification levels of ZBED3 in Huh7 cells, whereas NSUN5 overexpression promoted ZBED3 expression. These findings provide further substantiation for the hypothesis that ZBED3 functions downstream of oncogenic NSUN5 to drive HCC development. Nevertheless, additional experiments are warranted to validate this conclusion.

ZBED3 is considered a vital component of the Wnt/β-catenin signaling pathway, primarily due to its axin-binding activity [27]. However, conflicting evidence exists concerning ZBED3’s influence on Wnt/β-catenin signaling. Xiangbin Ruan et al. elucidated the indispensable role of ZBED3 in regulating neuronal layer fates during brain development through the Wnt/β-catenin pathway [28]. Farong Ou et al. demonstrated that ZBED3 activates this pathway, promoting chondrogenesis, a process further augmented by the lncRNA ZBED3-antisense 1, owing to its chromatin-binding ability [29]. In contrast, Haoying Xu reported that ZBED3 promotes adipogenic differentiation of adipose-derived mesenchymal stem cells, potentially achieved by negative regulation of the Wnt/β-catenin pathway [30]. The contradictory roles of ZBED3 in modulating Wnt/β-catenin signaling necessitate further investigation.

Notably, ZBED3 also participates in the development of various cancer types, with lung cancer being the most comprehensively investigated. In lung cancer, ZBED3 exhibits significant upregulation in tumor tissues compared with normal tissues. Furthermore, ZBED3 displays significant correlations with various clinical parameters, including enhanced lymph node metastasis, advanced TNM stages, elevated Ki67 expression, and unfavorable clinical outcomes [31, 32]. Mechanistically, ZBED3 exerts its influence by binding to axin, thereby suppressing the axin/adenomatous polyposis coli/glycogen synthase kinase-3β complex. This complex negatively modulates β-catenin levels, consequently impeding the Wnt/β-catenin signaling pathway [32, 33]. Additionally, ZBED3 promotes lung cancer cell proliferation by regulating the expression of proliferating cell nuclear antigen [34]. However, in gastric cancer, ZBED3 overexpression has been found to alleviate the malignant phenotype, suggesting a tumor-suppressing role [35]. Furthermore, an established prognostic model for colorectal cancer, incorporating eight genes, including ZBED3, revealed a negative correlation between ZBED3 expression and clinical risk scores [36]. These discordant findings underscore the distinct roles of ZBED3 in different cancer types. Unfortunately, to date, no comprehensive study has systematically elucidated the role of ZBED3 in HCC development.

Our experiments indicated that overexpressing ZBED3 reinstates the tumor-suppressive effects observed upon NSUN5 knockdown in Huh7 and Hep 3B cells. Additionally, since GSEA revealed that NSUN5 plays a role in the Wnt signaling pathway, where ZBED3 serves as a crucial mediator, we assessed the expression levels of key signaling molecules, such as β-catenin, c-Myc, and AXIN1 [37, 38]. Consequently, we observed a close association between NSUN5-associated tumorigenesis and the Wnt/β-catenin pathway.

Importantly, the elucidation of the roles of NSUN5 and its downstream molecule, ZBED3, in HCC development provides valuable insights for clinical applications. The prognostic significance of NSUN5 was substantiated by TCGA database. However, the potential utility of ZBED3 and other constituents of the Wnt/β-catenin signaling pathway as prognostic indicators requires further investigation. Additionally, NSUN5 and ZBED3 were identified as potential targets of antitumor drugs. Nonetheless, our study has a few limitations. Firstly, due to constraints in time and resources, we did not explore the other plausible candidates among the seven recognized NSUN5-associated genes. Secondly, the potential of ZBED3 as a prognostic marker and a tool for early HCC detection was not explored. Thirdly, we conducted in vitro experiments using HCC cell lines; bolstering the credibility of our findings would require additional in vivo experiments involving primary HCC cells and other HCC cell lines as well. In conclusion, we discovered that NSUN5 promotes HCC cell proliferation *via* the ZBED3/Wnt/β-catenin signaling pathway and could serve as a prognostic tool for patients with HCC. Our findings introduce a novel therapeutic target for HCC treatment.

## Conclusion

We elucidated that NSUN5 facilitates HCC development by targeting the ZBED3/Wnt/β-catenin signaling pathway.

## Materials and methods

### Online databases and data processing

A pan-cancer analysis was performed to gauge NSUN5 expression levels using clinical patient data acquired from TCGA database (https://cancer.gov/tcga). The dataset included comprehensive information for patients with HCC (LIHC, *n* = 371), encompassing clinical parameters such as grade, stage, TP53 mutation status, age, gender, and race. Statistical analysis was performed based on log_2_-transformed transcripts per million values. The prognostic significance of NSUN5 expression was determined through survival analysis utilizing Kaplan–Meier Plotter (https://kmplot.com/analysis/), along with the computation of HR and log-rank p-values. Patients with HCC were stratified into two groups based on NSUN5 expression levels, and their OS, RFS, PFS, and DSS were compared.

### Cell culture and stable knockdown/overexpression of NSUN5

Huh7 and Hep 3B cells (Chinese Academy of Sciences, Shanghai, China), free of mycoplasma contamination, were cultured in Dulbecco’s Modified Eagle’s Medium (Gibco, New York, USA) supplemented with 10% fetal bovine serum and 1% antibiotics (Sigma, USA). These cells were analyzed subsequent to NSUN5 knockdown and overexpression.

Endogenous NSUN5 knockdown was achieved using lentiviral vectors containing three distinct shRNAs: shNSUN5-1 (5′-GCAACTTCCAGAACGTGAA-3′), shNSUN5-2 (5′-GCAATAAGACCAGTCACTT-3′), and shNSUN5-3 (5′-GGCTTCTTCGTTGCTGTAA-3′). Exogenous NSUN5 overexpression was accomplished by employing the pCDH-CMV-GFP-puro plasmid in accordance with the manufacturer’s instructions (Yibaike Biotechnology, Beijing, China). The NSUN5 sequence utilized is provided in Supplementary Table 1. The efficacy of both knockdown and overexpression was assessed using quantitative reverse transcription–PCR (qRT-PCR) and western blotting.

### Western blotting

The expression levels of certain proteins in target cells were examined through western blot analysis. Cells were lysed in radioimmunoprecipitation assay buffer and then combined with sodium dodecyl sulfate-polyacrylamide gel electrophoresis sample buffer. Subsequently, electrophoresis was performed, and the protein bands were transferred onto polyvinylidene fluoride membranes (Bio Rad, USA). Following the transfer, the membranes were subjected to blocking and antibody incubations. Enhanced chemiluminescence was employed for the visualization of the protein bands, and imaging was performed using a western blot imaging system.

Antibodies used in Western blotting include: mouse anti-NSUN5 antibody (1:1000, Santa Cruz, CA, USA, catalog number: sc-376147), rabbit anti-ZBED3 antibody (1:1000, Bioss, Beijing, China, catalog number: bs-13553R), rabbit anti-AXIN1 antibody (1:1000, Cell Signaling Technology, Danvers, MA, USA, catalog number: 2087 S), rabbit anti-c-Myc-antibody (1:1000, Abcam, USA, catalog number: ab32072), rabbit anti-β-catenin (1:1000, Abcam, USA, catalog number: ab32572), mouse anti-GAPDH (1:2000, Abcam, USA, catalog number: ab8245).

### qRT-PCR

To evaluate the expression level of NSUN5 mRNA in HCC cell lines, we performed qRT-PCR. Total RNA was extracted using the Qiagen RNeasy Mini Kit (QIAGEN, Germany), following the manufacturer’s instructions. The relative expression of NSUN5 mRNA was determined using the ABI7500 Fast PCR instrument (ABI, Thermo Fisher). Glyceraldehyde-3-phosphate dehydrogenase served as the internal control, and the 2^-ΔΔct^ method was employed for quantifying relative gene expression. The primer details can be found in Supplementary Table 2.

### Colony formation assay

In both the experimental and control groups, single-cell suspensions were prepared and subsequently seeded in 60-mm Petri dishes (1000 cells per dish). The culture medium was replaced every three days. Once a substantial number of single clones exhibited more than 100 cells, they were fixed and stained. Three independent experiments were conducted for each group of cells to facilitate statistical analysis.

### EdU assay

A total of 1 × 10^5^ cells were incubated with EdU (Ribobio, Guangzhou, China) for 2 h in 96-well plates, fixed using 4% paraformaldehyde (Beyotime, Shanghai, China) for 30 min, stained using an EdU kit, and subsequently photographed. The EdU-positive rate was then calculated.

### MeRIP-seq and associated bioinformatic analysis

MeRIP-seq (m^5^C-seq) was performed by Cloudseq Biotech Inc. (Shanghai, China) following the manufacturer’s guidelines. Briefly, RNA samples were fragmented to yield a distribution of fragment sizes centered around ~200 nt. A total of 300 ng of fragmented mRNAs were designated as the Input material. In each reaction, fragmented RNAs were incubated with 1 µL of m^5^C antibody loaded onto magnetic beads in an IP buffer (containing 150 mM NaCl, 0.1% NP-40, and 10 mM Tris-HCl at pH 7.4) for 1 h at 4 °C. Subsequently, the target RNAs were purified using the RNeasy MiniElute Kit (Cloudseq Biotechnology, Shanghai, China). Both the Input and IP samples were subjected to 150-bp paired-end sequencing using an Illumina HiSeq sequencer.

Paired-end reads were obtained and subjected to quality control using Q30. Subsequently, the 3′ adaptor was trimmed, and low-quality reads were filtered out using Cutadapt software (v1.9.3). The processed reads were then aligned to the UCSC MM10 reference genome using HISAT2 (v2.0.4). Methylated RNA peaks were detected using the MACS software. Differentially methylated sites on RNAs were identified using diffReps. Finally, peaks identified by both software tools, which also exhibited overlap with mRNA exons, were discerned using custom-made scripts.

### Construction of ceRNA networks

MiRanda [39] and TargetScan [40] were employed to predict interactions between NSUN5 and miRNAs, leveraging the transcription profiles of shNC- and shNSUN5-treated Huh7 cells. The top five miRNAs were selected for subsequent analysis of their interactions with downregulated lncRNAs or circRNAs. For each miRNA, a maximum of five lncRNAs or circRNAs were identified. Subsequently, Cytoscape [41] was used to construct a ceRNA network.

### RIP-seq and associated bioinformatic analysis

mRNAs extracted from 5 × 10^7^ Huh7 cells were subjected to RIP following a previously described protocol with minor modifications. Briefly, the extracted mRNAs were incubated with an anti-NSUN5 antibody (mouse, 1:1000, Santa Cruz, CA, USA, catalog number: sc-376147) for Immunofluorescence (IP) using A/G magnetic beads. For control group, mouse IgG (1:1000, Beyotime, Shenzhen, China, catalog number: A7028) was used. The resultant RNAs were subsequently purified through treatment with proteinase K and TRIzol reagents before being subjected to bioinformatic analysis, as previously described. Briefly, Illumina HiSeq-generated reads were subjected to quality control measures. To enhance reproducibility, we performed correlation analysis among samples belonging to the same experimental group. Subsequently, gene peaks were analyzed in terms of peak width (the length of the protein-binding sequence), enrichment level, statistical significance, number of summit points, motif characteristics (identified using HOMER software), and genomic location. Furthermore, we performed GO and KEGG enrichment analyses of genes overlapping with peaks. These overlapping genes were defined as those exhibiting at least one peak at any genomic position.

### Tumor xenograft

Six-week-old male BALB/c nude mice were procured from the Zhejiang Academy of Medical Sciences and were randomly allocated into two groups, each comprising six mice. The Huh7 cell line was employed in this experiment. A total of 2 × 10^6^ stable shNC cells or stable shNSUN5 cells, diluted in 100 µL of Phosphate Buffered Saline, were injected into each mouse. Tumor volume was assessed weekly over a span of three weeks, followed by euthanization of the mice and measurement of tumor weights. Investigators were not blinded to the group allocation. The mouse experiments were conducted in accordance with the guidelines and regulations of the Zhengzhou University Institutional Animal Care and Use Committee.

### Statistical analysis

Spearman correlation analysis was conducted to evaluate the correlation between two variables, while a Student’s *t*-test was performed to compare two variable groups. Statistical analysis was performed using SPSS (v26) and the R package. Significance was determined at a two-sided *p*-value < 0.05.

### Supplementary information

Supplementary Table 1

Supplementary Table 2

Supplementary Figure 1

Supplementary Figure 2

Supplementary Figure 3

Supplementary Figure legends

## Data Availability

All datasets associated with this study are available from the corresponding author upon reasonable request. m^5^C-MeRIP-seq data are available in GEO DataSets GSE249809.
