# Supplementary material for: RNA 5-methylcytosine writer NSUN5 promotes hepatocellular carcinoma cell proliferation via a ZBED3-dependent mechanism
Source: Oncogene. 2024 Jan 5;43(9):624–35. doi: 10.1038/s41388-023-02931-z (PMC10890930; doi:10.1038/s41388-023-02931-z)
Supplement: Supplementary file 1 — Supplementary Table 1 [file 41388_2023_2931_MOESM1_ESM.docx]

**Supplementary Table 1. The information of NSUN5 sequence for overexpression.**

| **Gene name** | **Sequence (5'-3')** |  |
| --- | --- | --- |
| NSUN5 | atggggctgtatgctgcagctgcaggcgtgttggccggcgtggagagccgccagggctctatcaaggggttggtgtactccagcaacttccagaacgtgaagcagctgtacgcgctggtgtgcgaaacgcagcgctactccgccgtgctggatgctgtgatcgccagcgccggcctcctccgtgcggagaagaagctgcggccgcacctggccaaggtgctagtgtatgagttgttgttgggaaagggctttcgagggggtgggggccgatggaaggctctgttgggccggcaccaggcgaggctcaaggctgagttggctcggctcaaggttcatcggggtgtgagccggaatgaggacctgttggaagtgggatccaggcctggtccagcctcccagctgcctcgatttgtgcgtgtgaacactctcaagacctgctccgatgatgtagttgattatttcaagagacaaggtttctcctatcagggtcgggcttccagcctcgatgacttacgagccctcaaggggaagcattttctcctggaccccttgatgccggagctgctggtgtttcccgcccagacagatctgcatgaacacccactgtaccgggccggacacctcattctgcaggacagggccagctgtctcccagccatgctgctggaccccccgccaggctcccatgtcatcgatgcctgtgccgccccaggcaataagaccagtcacttggctgctcttctgaagaaccaagggaagatctttgcctttgacctggatgccaagcggctggcatccatggccacgctgctggcccgggctggcgtctcttgctgtgaactggctgaggaggacttcctggcggtctccccctcggatccacgctaccatgaggtccactacatcctgctggatccttcctgcagtggctcgggtatgccgagcagacagctggaggagcccggggcaggcacacctagcccggtgcgtctgcatgccctggcagggttccagcagcgagccctgtgccacgcactcactttcccttccctgcagcggctcgtctactccacgtgctccctctgccaggaggagaatgaagacgtggtgcgagatgcgctgcagcagaacccgggcgccttcaggctagctcccgccctgcctgcctggccccaccgaggcctgagcacgttcccgggtgccgagcactgcctccgggcctcccctgagaccacactcagcagtggcttcttcgttgctgtaattgaacgggtcgaggtgccaagctcagcctcacaggccaaagcatcagcaccagaacgcacacccagcccagccccaaagagaaagaagagacagcaaagagccgcagccggtgcttgcacaccgccttgcacatag | |
