# Supplementary material for: RNA 5-methylcytosine writer NSUN5 promotes hepatocellular carcinoma cell proliferation via a ZBED3-dependent mechanism
Source: Oncogene. 2024 Jan 5;43(9):624–35. doi: 10.1038/s41388-023-02931-z (PMC10890930; doi:10.1038/s41388-023-02931-z)
Supplement: Supplementary file 2 — Supplementary Table 2 [file 41388_2023_2931_MOESM2_ESM.docx]

**Supplementary Table 2. The information of primers sequences for qRT–PCR assay.**

| **Primer name** | **Sequence (5'-3')** |  |
| --- | --- | --- |
| GAPDH-F | GGAGCGAGATCCCTCCAAAAT | |
| GAPDH-R | GGCTGTTGTCATACTTCTCATGG |  |
| NSUN5-F | GGTGTACTCCAGCAACTTCCAGAAC |  |
| NSUN5-R | CCACTTCCAACAGGTCCTCATTCC |  |
| ZBED3-F | GGATGTGAGCCGCCGTGAGG |  |
| ZBED3-R | CAGGAGGACCTTTGTGATGACGCA |  |
