# Supplementary figures and images for: RNA 5-methylcytosine writer NSUN5 promotes hepatocellular carcinoma cell proliferation via a ZBED3-dependent mechanism
Source: Oncogene. 2024 Jan 5;43(9):624–35. doi: 10.1038/s41388-023-02931-z (PMC10890930; doi:10.1038/s41388-023-02931-z)

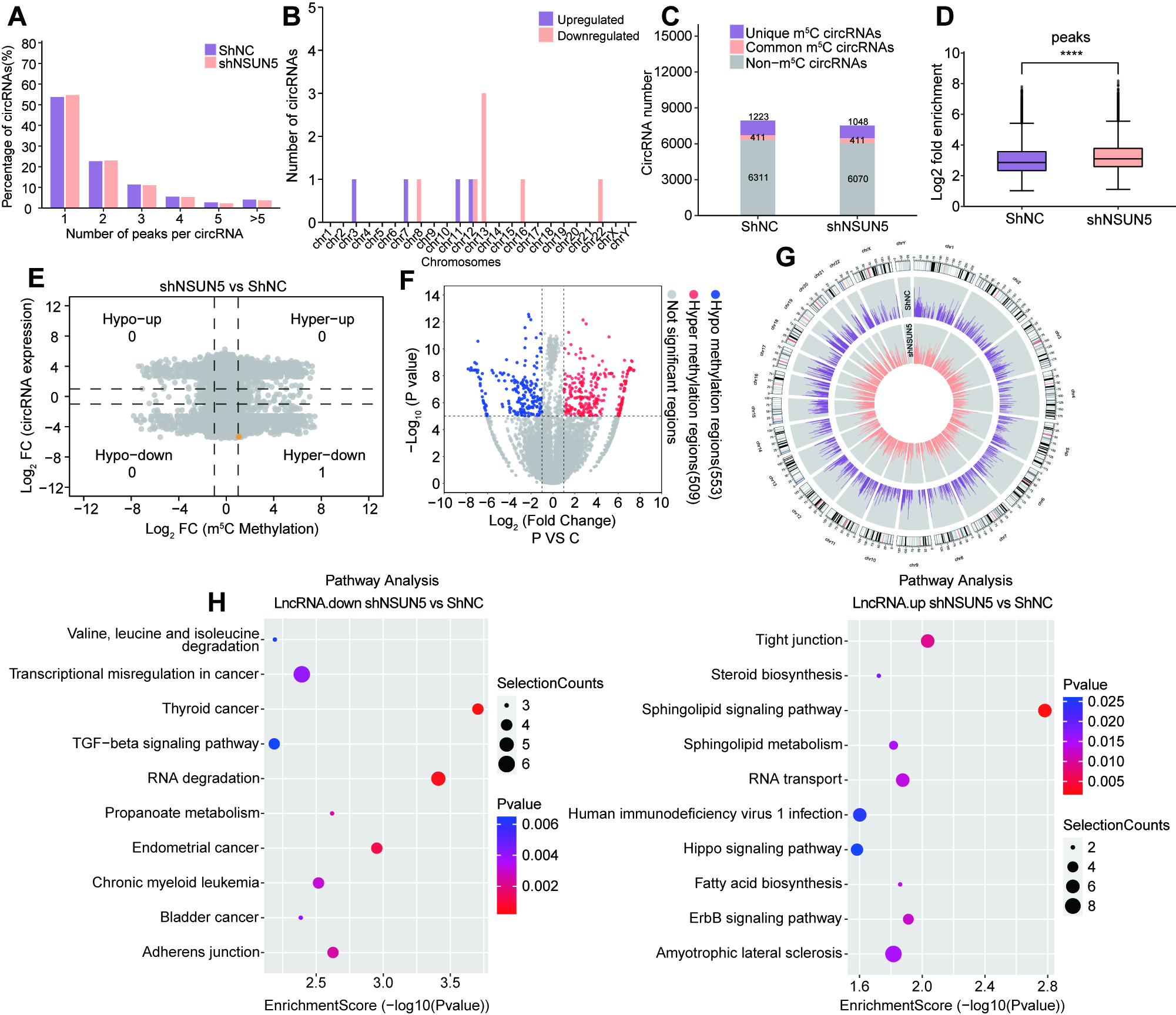

Supplement: Supplementary file 3 — Supplementary Figure 1 [file 41388_2023_2931_MOESM3_ESM.tif]

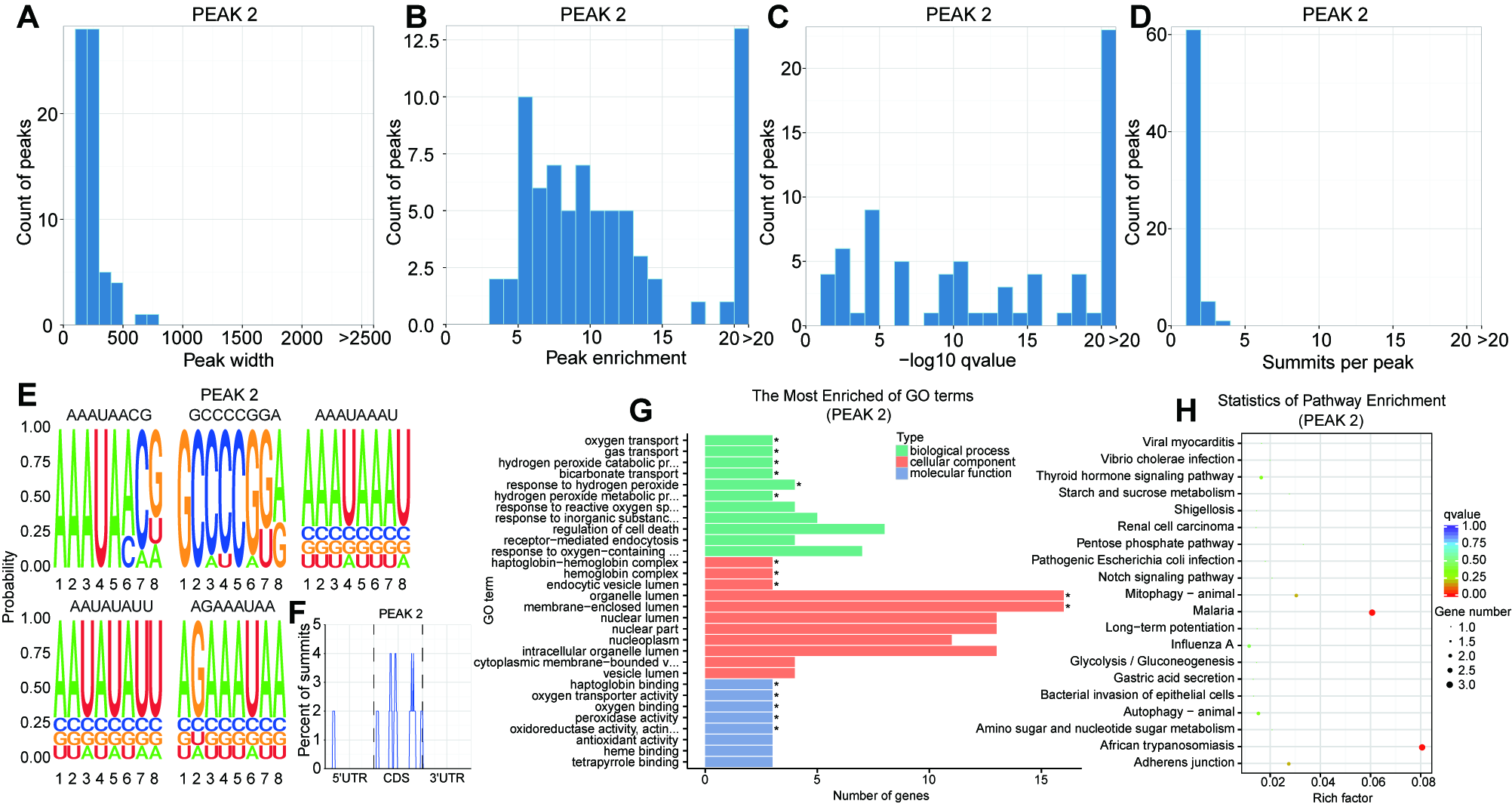

Supplement: Supplementary file 4 — Supplementary Figure 2 [file 41388_2023_2931_MOESM4_ESM.tif]

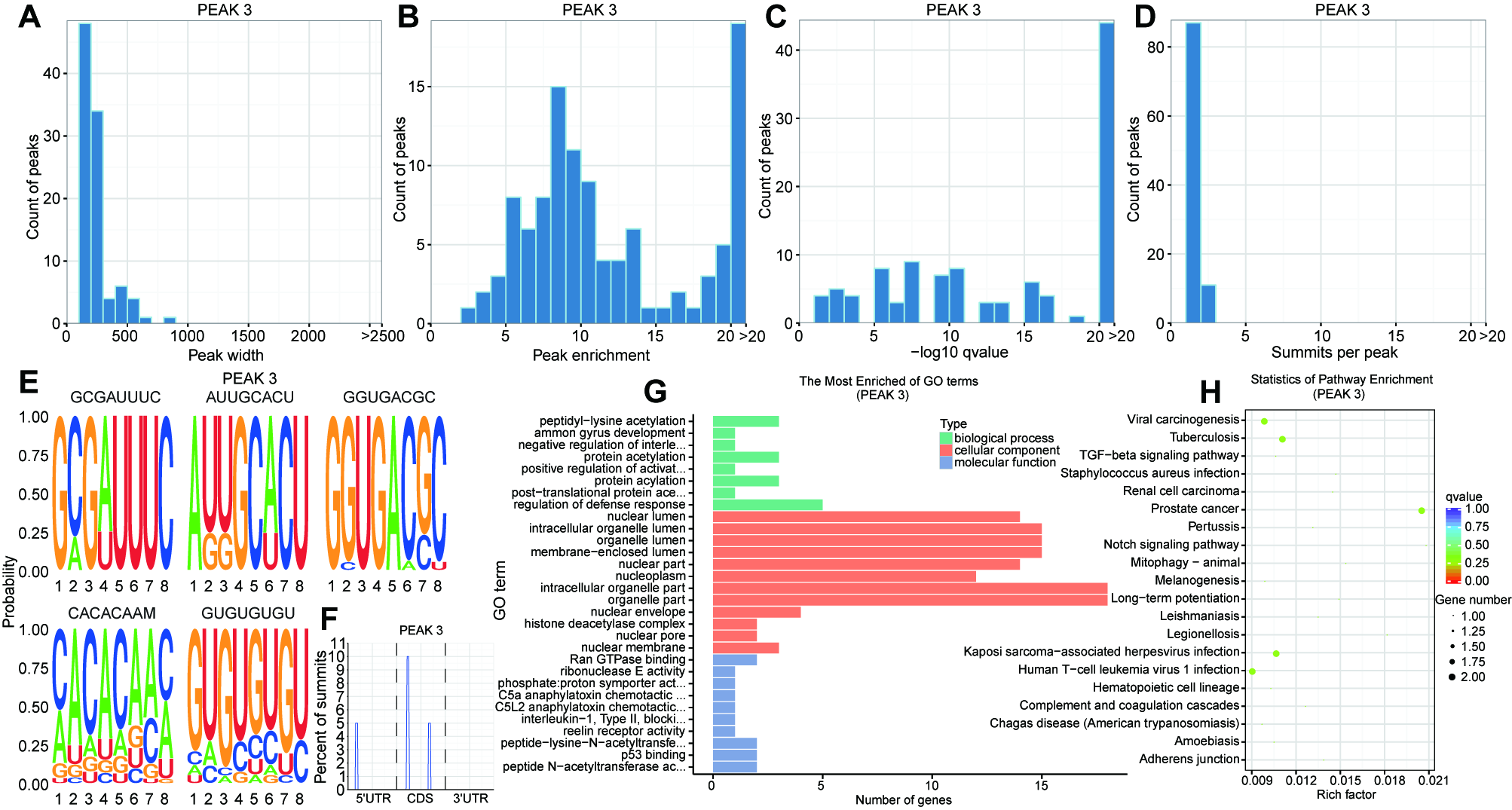

Supplement: Supplementary file 5 — Supplementary Figure 3 [file 41388_2023_2931_MOESM5_ESM.tif]
