# Supplementary material for: RNA 5-methylcytosine writer NSUN5 promotes hepatocellular carcinoma cell proliferation via a ZBED3-dependent mechanism
Source: Oncogene. 2024 Jan 5;43(9):624–35. doi: 10.1038/s41388-023-02931-z (PMC10890930; doi:10.1038/s41388-023-02931-z)
Supplement: Supplementary file 6 — Supplementary Figure legends [file 41388_2023_2931_MOESM6_ESM.docx]

**Supplementary Figure legends**

**Supplementary Figure 1. Results of m^5^C-MeRIP-seq of circRNAs in Huh7 cells with and without NSUN5 knockdown.** (A) Most circRNAs in both shNC- and shNSUN5-treated Huh7 cells exhibited a single m^5^C methylation peak, while some displayed multiple peaks. (B) Chromosomal distribution of upregulated and downregulated circRNAs. Chr3, Chr7, Chr11, and Chr12 exclusively displayed one upregulated circRNA each, whereas Chr8, Chr12, Chr16, and Chr22 exhibited only one downregulated circRNA. Additionally, Chr13 exhibited three downregulated circRNAs. (C) A total of 1,223 and 1,048 unique m^5^C-methylated circRNAs and 6,311 and 6,070 non-methylated circRNAs were identified in shNC- and shNSUN5-treated Huh7 cells, respectively. There were 411 m^5^C methylated circRNAs common to both groups. (D) The circRNA methylation peaks in both groups exhibited a significant difference in fold enrichment. (E) A four-quadrant diagram illustrated a single hyper-down circRNA following NSUN5 knockdown. No hyper-up, hyper-down, or hypo-up circRNAs were observed. (F) A volcano plot depicts 270 hypermethylated and 190 hypomethylated circRNA regions following NSUN5 knockdown. (G) A Circos map visualizes the chromosome locations and fold enrichment of m^5^C methylation peaks in both shNC- and shNSUN5-treated Huh7 cells. (H) Pathway analysis indicated that circRNAs associated with NSUN5 were predominantly enriched in thyroid cancer, RNA degradation, sphingolipid metabolism, and the tight junction pathway.

**Supplementary Figure 2. Results of RIP-seq of the Peak-2 group in Huh7 cells.** (A) The Peak-2 group predominantly consisted of RNAs with widths ranging from 100 to 300 nt, with no RNA peak exceeding 800 nt in width. (B) Peak enrichment analysis showed that most of the RNAs in the Peak-1 group exhibited total read counts ranging from 5 to 13. (C) Significance levels among different RNA peaks exhibited a scattered distribution. (D) Nearly all RNA peaks featured a single summit, with only a few displaying two to three summits. (E) The top five most significant mRNA motifs are illustrated. (F) The peaks exhibited enrichment within the CDS of mRNAs. A comparatively lower number of gene peaks were located in the 5′-UTR of mRNAs, while no gene peaks were observed in the 3′-UTR. (G) GO enrichment analysis showed that the genes overlapping with the peaks in the Peak-2 group were enriched in biological processes such as the regulation of cell death, cellular components, including organelle lumen, and molecular functions such as haptoglobin binding. (H) KEGG enrichment analysis indicated that the genes overlapping with the peaks in the Peak-2 group were significantly enriched in African trypanosomiasis, malaria, the pentose phosphate pathway, and the mitophagy-animal pathway.

**Supplementary Figure 3. Results of RIP-seq of the Peak-3 group in Huh7 cells.** (A) The Peak-3 group predominantly consisted of RNAs with widths ranging from 100 to 300 nt, with no RNA peaks exceeding 900 nt in width. (B) Peak enrichment analysis revealed that most of the RNAs in the Peak-1 group exhibited a total read count ranging from 5 to 14. (C) Significance levels among different RNA peaks exhibited a scattered distribution. (D) Nearly all RNA peaks exhibited a single summit, with only a few displaying two summits. The top five most significant mRNA motifs are illustrated. (F) Peaks displayed enrichment within the CDS of mRNAs. A relatively lower number of gene peaks were located within the 5′-UTR of mRNAs, while no gene peaks were observed in the 3′-UTR. (G) GO enrichment analysis showed that genes overlapping with the peaks in the Peak-3 group were enriched in biological processes such as embryonic development, cellular components, including intracellular organelles, and molecular functions involving binding to Ran GTPase. (H) KEGG enrichment analysis indicated that genes overlapping with the peaks in the Peak-3 group were significantly enriched in the Notch signaling pathway, prostate cancer, legionellosis, and the mitophagy-animal pathway.
